# The Development of Fluorescence Intensity Standards

**DOI:** 10.6028/jres.106.015

**Published:** 2001-04-01

**Authors:** A. K. Gaigalas, Li Li, O. Henderson, R. Vogt, J. Barr, G. Marti, J. Weaver, A. Schwartz

**Affiliations:** National Institute of Standards and Technology, Gaithersburg, MD 20899-8312; Center for Disease Control, Atlanta, GA; CBER, FDA, Bethesda, MD; CDER, FDA, Laurel, MD; Center for Quantitative Cytometry, San Juan, Puerto Rico

**Keywords:** fluorescence intensity, quantitative fluorescence, standards

## Abstract

The use of fluorescence as an analytical technique has been growing over the last 20 years. A major factor in inhibiting more rapid growth has been the inability to make comparable fluorescence intensity measurements across laboratories. NIST recognizes the need to develop and provide primary fluorescence intensity standard (FIS) reference materials to the scientific and technical communities involved in these assays. The critical component of the effort will be the cooperation between the Federal laboratories, the manufacturers, and the technical personnel who will use the fluorescence intensity standards. We realize that the development and use of FIS will have to overcome many difficulties. However, as we outline in this article, the development of FIS is feasible.

## 1. Introduction

Fluorescence-based assays have become important in health and research. Fluorescence assays have an advantage over other techniques in terms of increased sensitivity, selectivity, and simplicity of use. The majority of cellular assays require high levels of sensitivity to measure probe molecules that bind to their membranes. Probe molecules that bind to cells are designed to provide specific information about cell populations. For example, monoclonal antibodies that are directed against a specific cluster of determination (CD) antigen may provide positive identification of a specific subpopulation of biological cells. Furthermore, being able to quantify the number of those binding antibodies can give insight as to the level of maturation, activity, or pathology of the specific cell population [1]. In general, such insights provide basic understanding relating to the condition and functional biology of the cells. Such biological insight in a clinical environment can be valuable with respect to the diagnosis and prognosis of patients. Fluorescence is the most commonly used detection modality for DNA sequence analysis and for cytogenetic detection of chromosome alterations in human birth defects and cancer [2]. For example, accurate measurements for the HER2/neu oncogene are the basis for therapeutic decisions in some breast cancer and prostate cancer patients. Thus, accurate fluorescence quantitation is an important medical diagnostic issue for these genetic tests. An overview of the current state of fluorescence intensity quantitation can be found in the October 1, 1998 issue of *Cytometry* (Vol. 33, No. 2). There one will find a description of available fluorescence intensity standards [3], a summary of problems, a perspective on future developments [4, 5], and an example of fluorescence intensity quantitation [6].

**Fig. 1:**
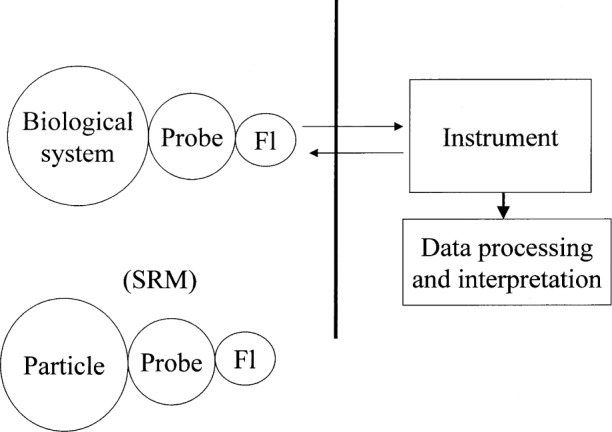
A schematic diagram of the entire fluorescence based biological assay. The word “probe” stands for a molecule that gives biological selectivity and the symbol “Fl” represents the fluorophore that is conjugated to the probe. The instrument and data processing software detects and processes the fluorescence measurements. The proposed particle SRM would be Fl conjugated to a probe-particle which would mimic the response of Fl in the biological assay. The comparison of the response of the SRM and biological assay would yield a quantitation of the fluorescence intensity.

A general procedure in fluorescence-based assays is to conjugate a fluorophore to some probe and use the fluorescence as an indicator of the presence of the probe molecules in the biological assay [7]. In addition to the advances in fluorophore conjugation chemistry, special instrumentation has been developed to detect these fluorescent probes, for example, flow cytometers [8] and digital fluorescence microscopy and image analysis. The data derived from these instruments are multi-parameter (there may be several fluorescence intensity signals and several scattering signals) and require sophisticated software to present the data in a meaningful manner, for example, histograms, color coded dot plots, and contour plots. The biological information being sought is dependent on all of these different factors as illustrated in Fig. 1, which shows the relation of all the components of a fluorescence-based biological assay. Clearly, there is a need for standard materials to verify the operation of the instrument such as wavelength standards and spectral correction standards [9]. However, at present there is no NIST Standard Reference Material™ (SRM™) available that would mimic the response of the conjugated fluorophore. It is the development and use of such a material (SRM™ in Fig. 1) that is the focus of this paper.

The majority of fluorescence-based biological assays measure fluorescence intensity and the results are dependent on the number of fluorescently labeled probes that bind selectively to the cells of interest in the assay (analyte) [10]. To obtain the number of probe molecules binding to the analyte, the measured fluorescence intensity is compared to the fluorescence intensity from a set of particles, each with a known number of fluorophores. This set of particles constitutes the fluorescence intensity standard (FIS). At first sight, it seems a simple matter to produce a FIS, namely, make a set of particles with different amounts of the fluorophore that is used in the biological assay. However, the complex nature of the fluorescence process makes this path more difficult than it appears. In short, the fluorescence from fluorophores attached to probes, which in turn are attached to analytes may be very different from fluorescence of the same fluorophore in solution or immobilized on particles. The fluorescence of a fluorophore is not an isolated molecular property, but depends to a high degree on the microenvironment of the molecule. Thus the production of fluorescence intensity standards that would be applicable to all assays even using the same specific fluorophore molecule is a complex issue. In the following we discuss the nature of fluorescence, its measurement, and propose some reference materials.

## 2. Properties of Fluorescence

**Fig. 2:**
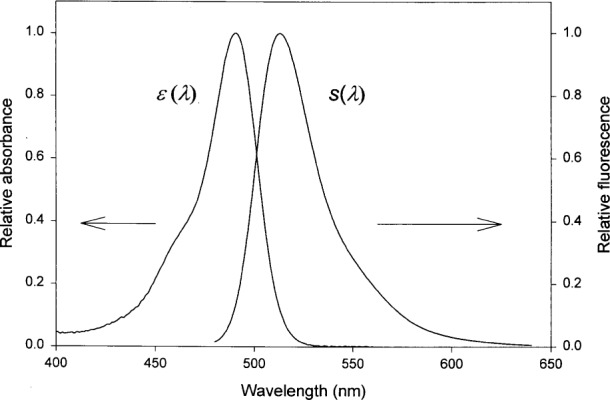
A plot of the wavelength dependence of the relative absorbance (excitation) and the relative emission(fluorescence) of fluorescein in Borate buffer with pH=9. The difference in the maximum wavelengths of the two plots is called the Stokes shift.

The properties of fluorescence have been described in many excellent books [11], here we provide only a summary of the facts pertinent to this work. The probability that a fluorophore will emit a photon is a product of the probability that a photon is absorbed and the probability that the excited state decays by emitting a photon (related to quantum yield). The probability of absorption is expressed by the molecular extinction coefficient, ***ϵ***(λ), m^2^/mol. The normalized (relative to the maximum absorption) molecular extinction coefficient of fluorescein is shown in Fig. 2. The important properties are the wavelength of maximum absorption and the spectral shape. Molecular extinction coefficients of fluorophore in aqueous solutions are somewhat sensitive to the microenvironment which tends to influence the energy of the electronic states. Thus changes in solution properties usually lead to shifts in the wavelength of maximum absorption and changes in the spectral shape. Figure 2 also shows the relative spectral function, *s*(λ), of the emitted (fluorescence) radiation when the excited state returns to the ground state. The important properties are the wavelength of maximum emission and the spectral shape. The initial excited electronic molecular state is in a sea of other excited states that become populated via various couplings to the initial state. As a consequence the de-excitation process has many possible paths many of which do not involve the emission of photons (non-radiative). The fluorophore microenvironment plays a crucial role in determining the de-excitation path and hence the quantum yield is a sensitive function of the microenvironment. Thus changes in solution properties usually lead to shifts in the wavelength of maximum emission and large changes in the quantum yield. The difference in the wavelengths of maximum absorption and maximum emission is called the Stokes shift. Fluorescein has a relatively small Stokes shift of approximately 20 nm, and as can be seen in Fig. 2 there is a substantial overlap between the absorption and emission spectra. Such an overlap leads to the possibility of resonant energy transfer between neighboring fluorophores providing yet another non-radiative path for de-excitation. This path (self-quenching) is important at high fluorophore concentrations.

## 3. Measurement of Fluorescence

The conceptual basis for measuring fluorescence intensity presented below is applicable to measurement techniques used by flow cytometers, spectrometers, and fluorescence imaging. Detailed description of the measuring process is available in the literature [12].

**Fig. 3:**
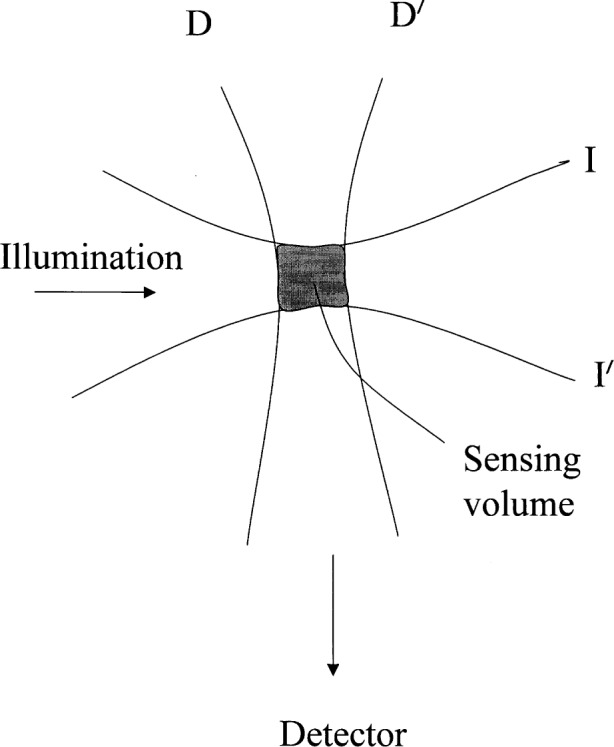
A schematic of the geometry of illumination and detection. The shaded volume is the sensing volume and is the volume over which the integral in Eq. (2) is taken. The sensing volume is the overlap of the illumination volume bounded by I and I′ and the detection volume bounded by D, D′. The geometry and various filters and optical elements give the instrumental factors in the fluorescence intensity measurements as described in Eq. (3).

Given a source of photons with known energies (spectral characteristics), the excitation optics will select a set of wavelengths that are incident on the sample. The excitation optics will illuminate a specific volume inside the sample (bounded by I and I′ in Fig. 3). The collection optics will collect light emitted from a volume element that we call the detection volume (bounded by D and D′ in Fig. 3). The total fluorescence intensity after the collection optics will depend on the overlap of the illumination and detection volumes and we call these overlapping volumes the sensing volume. Figure 3 is a schematic representation of the sensing volume. Only fluorophore molecules in the sensing volume can contribute to the fluorescence signal.

In addition, the fluorescence intensity depends directly on concentration of fluorophore in the sensing volume, molecular extinction coefficient, and the quantum yield. The collection optics will direct light of selected spectral range to the detector and provide a measurement of emission intensity over the selected range of wavelengths.

As a start, we define a simplified mathematical model of the detector output assuming that the detector is a photomultiplier tube (PMT). The current from the PMT will be given by

$$i_{F}=ge\int_{\lambda }Q(\lambda )E_{F}(\lambda )d\lambda$$ (1)

where *g* is the PMT gain, *e* is the elementary charge, *Q*(λ) is the quantum efficiency of the PMT photocathode at wavelength λ, and *E*_F_ (λ) is the flux of fluorescence photons per unit wavelength at the wavelength λ. The PMT current is a sum of fluorescence photons of all transmitted wavelengths. The fluorescence flux per unit wavelength from the sensing volume is given by

$$E_{F}(\lambda )\varepsilon c\phi (\lambda )T(\lambda )\int I_{o}(r)\Omega (r)dr$$ (2)

where the integral is carried out over the sensing volume defined in Fig. 3. *E*_F_(λ) is the number of photons of fluorescence of wavelength λ per second per nanometer, ***ε*** is the molar extinction coefficient (m^2^/mol) at the excitation wavelength, *c* is the concentration of the fluorophore (mol/L), ***ϕ*** (λ) is the probability per wavelength of emitting a photon at λ, *I*_0_(***r***) is the photon flux at ***r***(m^−2^s^−1^), and *T*(λ)***Ω***(***r***) is the probability that a photon emitted at position ***r*** will arrive at the PMT photocathode (assume that filter characteristics are the same for all paths through the collection optics). The factor *T*(λ) represents the filter characteristics and ***Ω***(***r***) represents the apertures and lenses of the collection optics. There are simplifications and omissions in Eq. (2) (e.g., polarization is not considered [13], we neglect the attenuation of incident light due to absorption by the sample, neglect lifetime). However, Eq. (2) should suffice for further discussion. Later, all the necessary details [14, 15] can be filled in to obtain working equations without changing the conceptual framework developed below. In addition, consideration regarding phenomena such as energy transfer, quenching and photobleaching will not be addressed in this presentation.

The total fluorescence signal will be modeled by inserting Eq. (2) into Eq. (1). The combination gives the explicit relation between the concentration of fluorophores and the measured current at the PMT detector. It is the basis for interpreting the fluorescence signal in terms of a concentration of fluorophores. Making the definition $\Omega \equiv \int I_{0}(r)\Omega (r)dr$, we get

$$i_{F}=\left[ge\Omega \varepsilon \int_{\lambda }Q(\lambda )\phi (\lambda )T(\lambda )d\lambda \right]c.$$ (3)

To a very good approximation the function ***ϕ***(λ) can be written as a product of a constant ***ϕ*** which is called the quantum yield, and a normalized spectral function *s*(λ) which gives the relative amount of emitted photons at different wavelengths. The quantum yield is defined as the ratio of the total emitted fluorescent photons to the total number of absorbed photons. Inserting this representation of ***ϕ***(λ) in Eq. (3) we arrive at the final result

$$i_{F}=\left[ge\Omega \varepsilon \phi \int_{\lambda }Q(\lambda )s(\lambda )T(\lambda )d\lambda \right]c.$$ (4)

The relation between the fluorescence signal, *i*_F_, and the fluorophore concentration, *c*, contains instrumental factors [*g*, *Q*(λ), ***Ω***, *T*(λ)] and molecular factors [***ϵ***, ***ϕ***, *s*(λ)]. To obtain consistent fluorescence intensity measurements across assays and instruments, these factors must be either controlled with appropriate correction coefficients applied to normalize the resulting measurements, or they must be eliminated from consideration in the measurement. To illustrate these points, we will discuss the following hypothetical measurements.

Suppose we have a set of standard solutions each with a different amount of a given fluorophore. The standard solutions define a relationship between the fluorescence intensity and the concentration of the fluorophore which is given by

$$i_{\text{F,S}}=\left[ge\Omega \varepsilon_{S}(\lambda_{\text{ex}})\phi_{S}\int_{\lambda }Q(\lambda )s_{S}(\lambda )T(\lambda )d\lambda \right]c_{S}$$ (5)

where the subscript S denotes quantities associated with standard solutions. The measured fluorescence intensities corresponding to the various standard solutions and the corresponding fluorophore concentrations can be displayed on a graph with the fluorescence intensity signal measured by the user along the vertical axis and the fluorophore concentrations of the standards along the horizontal axis [16]. (The slope of this calibration curve as modeled by the term in brackets in Eq. (5), will be different for different users because the instrumental factors will be different.) Next, using the same apparatus, we measure the fluorescence intensity from a sample of a test solution with unknown concentration of fluorophore. For the test (or analyte) solution the relationship between the fluorescence intensity and the fluorophore concentration is given by

$$i_{\text{F,T}}=\left[ge\Omega \varepsilon_{T}(\lambda_{\text{ex}})\phi_{T}\int_{\lambda }Q(\lambda )s_{T}(\lambda )T(\lambda )d\lambda \right]c_{T}$$ (6)

where the subscript T denotes quantities associated with the test solution. The instrumental factors in Eq. (6) are the same as in Eq. (5) since all measurements (standards and test) were performed with the same instrument and instrument settings. Furthermore, suppose we ensure that the microenvironment in the test solution is the same as the microenvironment in the standard solutions, then the quantum yields and spectral functions will be identical and the terms in the brackets in Eq. (5) and Eq. (6) will be identical. Thus the ratio of Eq. (5) and Eq. (6) reduces to the ratio of measured currents on the left side of the equal sign and the ratio of corresponding fluorophore concentrations on the right side of the equal sign. The equality of the two ratios suggests that the calibration curve obtained with the standard solutions can be used directly to obtain the fluorophore concentration of the test solution. The ratio of the terms in the brackets in Eq. (5) and Eq. (6) will equal one for all instruments, hence measurements carried out on different instruments will give the same concentration of fluorophore in the test solution. The above measurement is the ideal situation, it is achieved under the following conditions. First, the measurements of the solution standards and test solution are performed under the same instrument settings and conditions, i.e., the same *g*, ***Ω***. Second, the molecular properties (the excitation spectrum ***ϵ***(λ_ex_), the emission spectrum, *s*(λ), and quantum yield, ***ϕ*** of the fluorophore in the standards and the tests solutions are identical.

In a second hypothetical case some of the molecular properties of the fluorophores in the test and standard solutions may be different. Specifically we assume that the quantum yield is different but the emission spectral functions, *s*_S_(λ) and *s*_T_(λ), and the molecular extinction coefficients ***ϵ***_S_ and ***ϵ***_T_ are the same (standards and test solution are spectrally matched). Suppose we perform the same set of measurements as in the “ideal” case above and then use the calibration plot that was generated with the standards solutions to determine the unknown fluorophore concentration. The “concentration” obtained using this procedure will not correspond to the true concentration of fluorophores in the test solution. The ratio of the terms in Eq. (5) and Eq. (6) suggest that the true concentration *c*_T_ will be obtained from the relationship where $c_{T}=\phi_{S}/\phi_{T}\cdot c_{T}^{*}$ where $c_{T}^{*}$ is the concentration of the fluorophore in the test solution as determined using the calibration curve from the standard solutions. Thus, even in the case of solutions of the same fluorophore molecule, a single set of fluorescence intensity standards may not give the true concentration of fluorophore in arbitrary test solutions. Corrections based on changes of quantum yield may be needed. Since in practice it is very difficult to make quantum yield the same in standard and test solutions the majority of measurements of fluorescence concentration based on solution standards will give a concentration in terms of an equivalent number of soluble fluorophores. The above discussion motivates the rationale for adopting the units of fluorescence intensity referred to as MESF (molecules of equivalent soluble fluorophore). However even when talking about MESF units there are other issues such as spectral matching that are important for the proper use of fluorescence intensity standards. These issues are discussed in the following.

## 4. Achieving Instrument Independent Measurements

In practice it is very difficult to achieve the conditions necessary for the ideal measurement of fluorescence intensity. The sensitivity of the quantum yield to the fluorophore microenvironment variability is well established making quantum yield a difficult property to match. A practical solution has been the definition of the MESF unit such that the fluorophore “concentration” in a test solution is determined using a calibration with fluorophores in standard solutions. The ratio of quantum yields of the fluorophores in the standard and test solutions serves as a correction factor (as discussed above). In many cases it is sufficient to know that the measured fluorophore concentration is proportionate to the true concentration, so that MESF units are a practical alternative. However even measurements of fluorescence intensity in terms of MESF units are not without problems. A major problem is the dependence of the resulting MESF assignment on instrument properties. In the following we discuss the conditions which have to be met in order to achieve instrument independent measurements of fluorescence intensity in units of MESF.

**Fig. 4:**
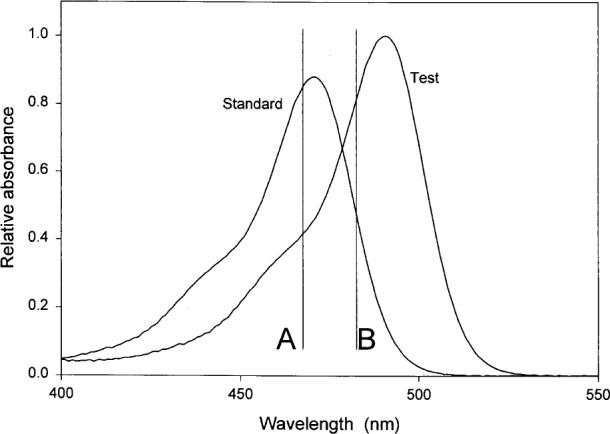
A graph of hypothetical excitation profiles of fluorophores in the standard and test solutions. The vertical lines represent the excitation wavelengths used by two different instruments called A and B. The ratio of the molar extinction coefficients of the standard to test solutions is greater than 1 for instrument A and less than 1 for instrument B. The difference in the ratio would lead to different determinations of the concentration of the fluorophore in the test solution.

**Fig. 5:**
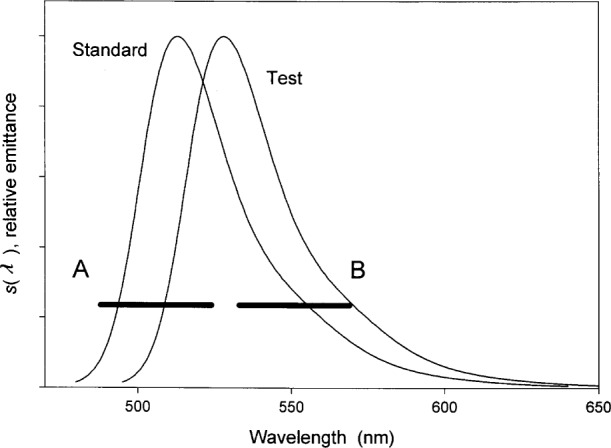
A graph of hypothetical emission spectral functions, *s*(λ), of a fluorophore in standard and test solutions. The thick horizontal lines represent the filter transmission range for instruments A and B. For the sake of simplicity the transmission will be set to 1. The ratio of the integral *s*(λ) of the standard and test solutions over the transmission range will be greater for instrument A than that for instrument B. The difference in the ratio would lead to different determinations of the concentration of the fluorophore in the test solution.

The first criterion is that the measurements of the fluorescence intensity of fluorophores in solution standards and fluorophores (analytes) in test solution must be performed with the same instrument under the same instrument settings and conditions, i.e., the same g, Ω. A second criterion is that the excitation spectrum ***ϵ***(λ_ex_) of the fluorophores in the standard and test solutions should match. Figure 4 illustrates the problem where the difference of the absorption spectra of the fluorophores in the standard and test solutions has been exaggerated on purpose. The vertical lines in Fig. 4 represent excitation wavelengths used by two different instruments called A and B. The ratio of the extinction coefficients of the standard and test solutions is greater than 1 for instrument A but less than 1 for instrument B. The different ratios will lead to different calibration lines for the two instruments resulting in different determination of the MESF concentration of fluorophores in the test solution. If the excitation spectra in Fig. 4 were identical then the ratio of the extinction coefficients would be 1 for both instruments. The practical issue is to determine how well the excitation spectra have to match to yield acceptable results. The third condition is that the emission spectra of the fluorophores in standard and test solution should match. Figure 5 illustrates this problem. Again the difference of the two emission spectra in Fig. 5 was exaggerated for purpose of illustration. The thick horizontal lines represent the transmission properties of the optical detection system in instrument A and B. For the sake of simplicity we will assume that the transmission is 1 in the range indicted by the horizontal lines in Fig. 5. Then the ratio of the integrals in Eq. (5) and Eq. (6) will be greater for instrument A than for instrument B. This difference in ratios will lead to a difference in determined fluorophore concentration by the two instruments. If the emission spectra are identical the ratio of the two integrals is always 1 and independent of the instrument. Again the practical issue is what is a sufficiently good match of the emission spectra?

In the previous discussion we implied that all measurements were performed in solutions. Most biological assays are performed on immobilized fluorophores. Furthermore the measuring instruments such as flow cytometers and microscopes are most conveniently calibrated using standards with fluorophores immobilized on particles [17–19]. The above discussion of solution measurements is directly applicable to measurements with fluorophores immobilized on particles and biological cells. The analyte intensity measured against standard particles can be expressed in terms of equivalent fluorescence. The results will be independent of instrument as long as the three criteria discussed above are met. This holds true even when the analytes and standard particles are suspended in an environment different than that used to calibrate the standard particles.

## 5. Additional Measurement Problems With Particles

Equation (4) provides the relationship between fluorescence intensity and fluorophore concentration under a set of assumptions one of which is the absence of self-quenching. The effective concentration of fluorophores immobilized on particles can sometimes reach levels where self-quenching becomes significant. In this case Eq. (4) is not a valid relation between measured fluorescence intensity and the number of labeled probes binding to particles or cells. Correction factors are needed which reflect any change in quantum efficiency as a function of labeling density of the fluorophore [20–22]. Another significant difference for immobilized fluorophores is the smaller depolarization ratio due to the constrains on rotational diffusion of the fluorophore.

## 6. NIST Primary Fluorescence Intensity Standards

### 6.1 Fluorophores in Solution

The first and most simple standard to be offered will be solutions with specified concentrations of fluorophore. For example, the standard could be a micromolar solution in a specific buffer at a specific pH. The concentration of fluorophores in the standard solutions would be intentionally set high so that users of the standards would have to dilute them in the buffers that they are using. The dilution would be an attempt to equalize the microenvironments of the fluorophores in the standard and test solutions and achieve the ideal measurement conditions as discussed above. Manufacturers who make secondary standards could use the NIST primary solutions to calibrate their spectrofluorometer and then assign values of fluorophore concentration to their secondary standards. These concentration assignments would be valid only when the manufacturer’s secondary solution standards are prepared under the same environment and conditions as the NIST solution standards. The solution standards could also be used to calibrate particles with immobilized fluorophores if the particles are suspended in the same buffer which is used to dilute the standard. Under these conditions the microenvironments of the fluorophore immobilized on the particles and in solution will most likely be sufficiently similar to insure spectral matching although the quantum yields will most likely differ. The particles would be calibrated in terms of MESF units. If needed, the ratio of quantum yields must be determined and used to adjust the concentration.

### 6.2 Fluorophores in Powder Form

The second standard that could be offered is a pure dry powder of the fluorophore. These powders would be certified as to purity, extinction coefficient and quantum yield under highly specified environmental conditions when made into solutions. Stability of these properties over time would also be specified. Protocols to obtain the specified fluorescence properties in solution would be supplied with the standards. The powder and solution formats each have their advantages. As we gain experience with the use of fluorescence intensity standards it may become apparent that one of the formats is preferred.

## 7. Fluorescence Intensity Standards for Cytometers

Fluorescent particle standards have been used in conjunction with specialized instrumentation, namely flow cytometers and fluorescence imaging microscopes. These instruments take readings from large numbers (1000 to 100 000) of individual cells and present the data in the form of histograms which are statistically analyzed. Thus, the important data to provide with a particle fluorescence intensity standard (FIS) is the average MESF value per particle along with the relative standard deviation of the MESF value distribution. The utilization of particle standards can be very different from that of solution standards. Particles are physical entities that can be removed from the suspension, washed and re-suspended in a different solution. Thus it is possible to assign calibration values to the standard particles in one solution and then use them in a different solution when assaying an analyte. The motivation for this is a desire to match the microenvironments of the fluorophores attached to the standard particles and the analyte particles (for example, cells in a flow cytometer). In what follows, we will focus on the initial assignment of the fluorescence intensity values to the NIST standard particles and the transfer of this calibration to secondary particle standards.

### 7.1 Fluorophores Immobilized on Particles

For practical purposes, polymeric microbeads have been used as the carrier particles for the fluorophores, since the microbeads may be manufactured in specific sizes with high uniformity. A major complication is the uncertain microenvironment of the fluorophore since it may be inside the microbead or bound close to the surface of the microbead. A spacer molecule can be used to maximize the sensitivity to solution while minimizing the sensitivity to particle surface. Self-quenching in conjunction with spectral shifts may become important if the density of immobilized fluorophores is large. The major advantage is that the particle FIS and cellular analytes will give similar fluorescence response in instruments such as flow cytometers if the solution conditions for both the particle standards and the cells are matched.

The fluorescence intensity units of the primary standard particles would be assigned at NIST with a spectrofluorometer utilizing the NIST FSI reference solutions. Since the quantum yields between solutions and immobilized fluorophores will most likely not be equal, we can not express the fluorescence intensity of the NIST particles in terms of actual numbers of fluorophore molecules bound to the particles. However, the intensity of the fluorescent particles can be expressed as Molecules of Equivalent Soluble Fluorophore (MESF). It must be noted that MESF “units” do not indicate the number of molecules of fluorophore attached to the particle, but rather the equivalent number of the fluorophore dissolved in the same media.

MESF units are assigned to microbead suspensions using a spectrofluorometer calibrated with a set of primary reference solutions of the same fluorophore. For a suspension of microbeads of number concentration, *c*_b_, the equivalent molar fluorescence can be directly converted to the average MESF units per particle by multiplying by Avogadro’s Number and dividing by the number concentration of particles in the measured suspension. It is important to note that MESF units are not general units like the meter. Rather, MESF units are fluorescence intensity units specific to the measured fluorophore, and therefore not comparable across fluorophores.

NIST primary FIS microbeads could serve as a calibration material for secondary particle standards. The application of the NIST microbeads to calibrate and assign fluorescence intensity values to secondary microbeads requires that the three criteria concerning spectral matching, equivalent microenvironments, and instrument settings be met. Under these ideal conditions, the proportionality of the intensities between the NIST standards and the secondary standards are constant. In the more likely case where the NIST reference microbeads and the secondary standard microbeads are not of the same material or do not use the same immobilization chemistry, the fluorophores immobilized on the two microbeads would have different quantum yields and possibly different spectral properties. Calibration of the secondary microbead standards in MESF units as assigned against the primary NIST microbead standards would have to consider a correction factor, if the three criteria for ideal measurements are not met. The use of the NIST microbeads would be attractive since all users would use the same MESF units and assign the calibration values to their secondary standards using a flow cytometer or fluorescence imaging instrument.

## 8. Conclusion

Three possible forms of primary fluorescence intensity standards have been proposed: solutions of fluorophores, pure powder, and particles with immobilized fluorophores. The choice of fluorophores to be developed in these formats should be dictated by the practical needs of the scientific community.

At present, the most widely used and established fluorophore in many diverse fields is fluorescein. It is our intent to use fluorescein as the model fluorophore for characterization, because it is sensitive to many of the environmental factors that must be considered in the general approach to fluorescence intensity measurements.

In the near future, we hope to offer an aqueous solution of fluorescein as an SRM™. The fluorescein reference solution will be a concentrated (~60 μmol/L) solution of fluorescein in 0.1 mol/L Borate buffer at 9.0 pH. To avoid the inherent difficulties of using volumetric units, the reference solution will be calibrated in mass units: grams of fluorescein per grams of buffer. The reference solution can be used to determine the concentration of fluorescein in arbitrary buffers. In practice, the reference solution will be diluted by 100, 200, etc., using the same buffer in which the unknown fluorescein analyte is found. Assuming the concentration of the buffer in the diluting solution is sufficiently high, the dilution will ensure that the reference fluorescein molecules and the test fluorescein molecules have the same microenvironment, thus yielding identical fluorescence properties, i.e., matching spectra and quantum yield. (We will test the assumption that a 100 or greater dilution with a sufficiently concentrated diluent buffer results in a buffer identical to the diluent.) The diluted reference solutions will provide a calibration of fluorescence intensity as a function of fluorescein concentration. This calibration may be used to determine the concentration of fluorescein in the test solution. A major use of the FIS solution standard will be in evaluating fluorescence reagents. In practice, most fluorescence reagents are used as solutions that are immobilized as the solution “stains” the solid phase (cell, microbead, or chip). These reagents must be first evaluated in their stock solutions, requiring comparison with a solution-based standard.

In preparation for a fluorescein particle SRM™, we are conducting research to characterize the differences between the fluorophore in solution and bound to a surface, e.g., spectra shift, change in quantum efficiency, change in extinction coefficient, etc. Given these differences, it is also important to report quantitative estimates of systematic errors introduced in the definition of the MESF units.

## References

[1] Marti G (1997). History, Practical Theory and Consensus of Quantitative Flow Cytometry Measurements, Standards for QC/QA in Flow Cytometry.

[2] Barker PE, Schwab M (1993). Junction mapping of translocation chromosomes by fluorescence in situ hybridization (FISH) and computer image analysis in human solid tumors. Methods of Molecular Genetics.

[3] Schwartz A, Marti GE, Poon R, Gratama JW, Fernandez-Repollet E (1998). Standardizing flow cytometry: A classification system of fluorescence standards used for flow cytometry. Cytometry.

[4] Gratama JW, D’Hautcourt JL, Mandy F, Rothe G, Barnett D, Janossy G, Papa S, Schmitz G, Lenkei R (1998). Flow cytometric quantitation of immunofluorescence intensity: Problems and perspectives. Cytometry.

[5] Lenkei R, Gratama JW, Rothe G, Schmitz G, D’Hautcourt JL, Arekrans A, Mandy F, Marti G (1998). Performance of calibration standards for antigen quantitation with flow cytometry. Cytometry.

[6] Iyer SB, Hultin LE, Zawadzki JA, Davis KA, Giorgi JV (1998). Quantitation of CD38 expression using QuantiBRITE (TM) beads. Cytometry.

[b7-j62gai] 7R. P. Haugland, Molecular Probes, Inc., 1999.

[8] Shapiro HM (1995). Practical Flow Cytometry.

[9] Velapoldi RA, Mielenz KD, Fluorescence A (1980). Standard Reference Material: Quinine Sulfate Dihydrate.

[10] Waggoner A (1995). Meth Enzymol.

[11] Lakowicz JR (1983). Principles of Fluorescence Spectroscopy.

[12] Mielenz KD, Mielenz KD (1982). Photoluminescence Spectrometry, Measurements of Photoluminescence.

[13] Cehelnik ED, Mielenz KD, Velapoldi RA (1975). Polarization Effects on Fluorescence Measurements. J Res Natl Bur Stand (US).

[14] Mielenz KD, Velapoldi RA, Mavrodineanu R (1977). Standardization in Spectrophotometry and Luminescence Measurements.

[15] Mavrodineanu R, Schultz JI, Menis O (1973). Accuracy in Spectrophotometry and Luminescence Measurements.

[16] Schwartz A, Fernandez-Repollet E, Vogt R, Grantama JW (1996). Standardizing flow cytometry: Construction of a standardized calibration plot using matching spectral calibrators. Cytometry.

[17] Brown MC, Hoffman RA, Kirchanski SJ, Andreeff M (1986). Controls for Flow Cytometers in Hematology and Cellular Immunology, Clinical Cytometry.

[18] Oonishi T, Uyesaka N (1985). A new standard fluorescence microsphere for quantitative flow cytometry. J Immunolog Meth.

[19] Visser J, Haaijman J, Trask B, Knapp W, Holubar K, Wick G (1978). Quantitative Immunofluorescence in Flow Cytometry.

[20] Vogt RF, Marti GE, Schwartz A, Tyrer HW (1994). Quantitative calibration of fluorescence intensity for clinical and research applications of immunophenotyping by flow cytometry. Cytometry.

[21] Schwartz A, Fernandez-Repollet E (1995). Technical aspects of fluorescence quantitative measurements by flow cytometry. Clin Immunolog Newslett.

[22] Schwartz A, Fernandez-Repollet E (1993). Development of clinical standards for flow cytometry. Ann N Y Acad Sci.

